# Experience with HCV Detection and Molecular Genetic Characterization among Otherwise Healthy Pregnant Women and Their Partners in the Republic of Guinea

**DOI:** 10.3390/microorganisms12101959

**Published:** 2024-09-27

**Authors:** Yulia V. Ostankova, Diana E. Reingardt, Alexandr N. Schemelev, Thierno A. L. Balde, Sanaba Boumbaly, Areg A. Totolian

**Affiliations:** 1 St. Petersburg Pasteur Institute, St. Petersburg 197101, Russia; dianavalutite008@gmail.com (D.E.R.); tvildorm@gmail.com (A.N.S.); totolian@pasteurorg.ru (A.A.T.); 2 Research Institute of Applied Biology, Kindia 100 BP 75, Guinea; thiernoamadoulabe.balde@gmail.com; 3 Centre International de Recherche sur les Infections Tropicales en Guinée, Nzerecore 400 BP, Guinea; drboumbaly@yahoo.fr

**Keywords:** hepatitis C markers, viral hepatitis C, hepatitis C virus, HCV variability, genotypes, subtypes, laboratory diagnostics, Republic of Guinea

## Abstract

According to recent data, there are currently 170 to 200 million people infected with HCV worldwide, and the number of new cases annually is approximately 40,000. Thus, the overall prevalence of the pathogen in the world is about 1.8–3%. The dynamic monitoring of circulating viral variants in specific groups that reflect the situation in the wider population, including potential pathogen spread, is of high importance for predicting the epidemiologic situation. Pregnant women are such a group. The Republic of Guinea is one of the poorest countries in the world, in which medicine receives little finance from the state. Among other conditions, HCV infection is not monitored in the country. This work used blood plasma from pregnant women living in the Republic of Guinea and their partners (1810 and 481). ELISA diagnostic kits were used to detect serologic markers, and PCR diagnostic kits were used to detect molecular biologic markers. Sanger sequencing, followed by phylogenetic analysis, was used for genotyping. The present study shows that HCV antibodies were detected in 3.2% of the pregnant women examined and in 3.33% of their male partners. HCV RNA was detected in 0.5% of cases in women and in all anti-HCV-positive male partners (3.33%). HCV RNA was more common in the men than in the pregnant women (χ^2^ = 25.6, df 1, *p* < 0.0001, RR = 6.69 with 95% CI: 2.97–15.04). The HCV viral load was determined for all the RNA-HCV-positive samples. The HCV viral load exceeded 1000 IU/mL in all nine women and only in two cases in men. The HCV genes *NS5A* and *NS5B* and the *NS3* gene fragment were sequenced for 11 samples. Subtype 2q was determined for three isolates and 2j for another three isolates. Another five isolates could not be confidently assigned a subtype because different results were obtained with different methods of analyzing the three viral regions. It can be assumed that these isolates belong to new viral subtypes or to recombinant forms between genotype 2 subtypes. No drug resistance mutations were identified, but a large number of natural polymorphisms in the analyzed genomic regions of the HCV isolates were shown. These results may serve as baseline data for the future planning of a nationwide estimate of the prevalence of bloodborne infections among pregnant women.

## 1. Introduction

Unlike many other infectious diseases, the burden of viral hepatitis has increased significantly in recent decades, and viral hepatitis is by now the seventh leading cause of death worldwide [1]. This includes a virus of global concern: the hepatitis C virus (HCV). An estimated 1.4 million deaths per year are attributable to acute viral hepatitis, as well as liver cancer and cirrhosis associated with chronic viral hepatitis. Of these deaths, approximately 48% are caused by HCV [2]. According to recent data, there are currently 170 to 200 million people infected with HCV worldwide, and the number of new cases annually is approximately 40,000. Thus, the overall prevalence of the pathogen in the world is about 1.8–3% [3]. The representation of the infection in different countries is uneven and depends on the geographical region. The lowest prevalence, 0.5–1.5%, is characteristic of Australia, the USA, and most European countries. It increases to 2.3% in the countries of Southeast Asia. The highest prevalence is shown in the countries of East and Central Asia and Africa, where it reaches 7.5% [4].

The molecular genetic variation of viruses can show a relationship with spatial and temporal changes, i.e., it can evolve over time, and it can spread in geographic regions, risk groups, or key populations, alongside changes in transmission routes [5]. Therefore, dynamic monitoring of circulating viral variants in population groups reflecting the situation in the population as a whole and potentially capable of spreading the pathogen is of high importance for predicting the epidemiologic situation. Pregnant women are such a population group, as they actually demonstrate the epidemiologic profile of the sexually mature heterosexual population of a particular geographic region under study and participating in the infection process. Understanding the epidemiologic features of HCV prevalence, as well as its genotypic and mutational profiles, in pregnant women in a given region is important because this information can be used to develop appropriate control and prevention measures. The timely and accurate laboratory diagnosis and, if necessary, treatment of pregnant women is vital to prevent mother-to-child transmission and improve control of socially significant diseases [6].

The Republic of Guinea is one of the poorest countries in the world, with a population of more than 13.6 million, located on the Atlantic coast of West Africa [7]. Healthcare is underfinanced by the state. For example, the health and social work sectors’ contribution to the gross domestic product to did not exceed 3.2% in 2019 [8]. Among other features, a significant reduction in services to prevent vertical transmission of HIV has been seen. These are key services for improving maternal and child health, and the average number of visits to prenatal care clinics has decreased [9,10]. It should be noted that in the Republic of Guinea, the most significant obstacles to monitoring the circulation of HIV, HBV and HCV, as well as clinically significant mutations of these pathogens, are not only limited financial resources, lack of high-tech equipment, and qualified specialists. There is also a lack of understanding of the importance of such information for epidemiological surveillance, the development of a strategy to prevent infection, and reducing the incidence and prevalence of bloodborne infections in the country.

The aim of this work was to be a pilot study of the prevalence of HCV markers in a cohort of pregnant women, allowing the identification of basic facts related to HCV prevalence in the Republic of Guinea, which is necessary for the planning of projects related to an in-depth study of the epidemiologic situation in Guinea.

## 2. Materials and Methods

The material for this study was blood plasma obtained from pregnant women and their partners. The material was collected from health facilities in the Republic of Guinea in 2020 with the consent of the subjects. HCV markers were detected in Guinea, and genotyping studies were performed in Russia at the St. Petersburg Pasteur Institute.

This work used blood plasma from 1810 pregnant women from six cities (Kindia, Conakry, Kankan, Farana, Macenta, Pita). Patient exclusion criteria for the study were a history of HIV, HBV, or HCV infection. 

**Figure 1:**
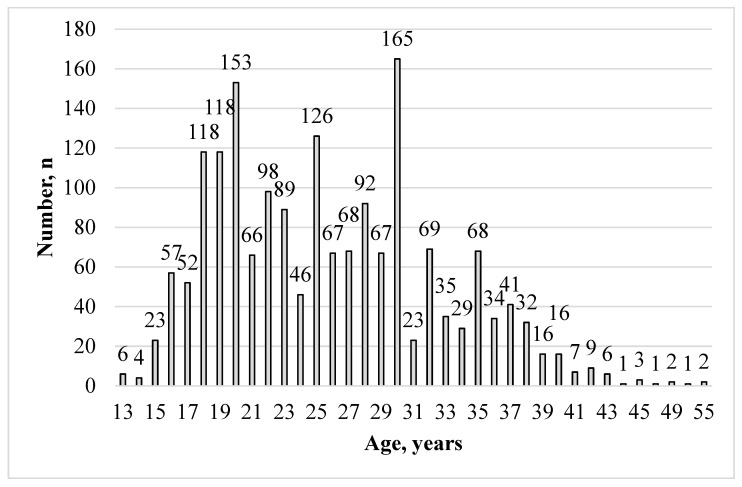
Age distribution of pregnant women in the study group.

The group of pregnant women was divided by age into subgroups: 13–17 years; 13–19 years; 18–35 years; and ≥36 years. In addition, a group of those 20–24 years old was defined, as it is known that adolescent girls aged 10–19 years are at higher risk not only of eclampsia and postpartum endometritis, but also of systemic infections, than young women aged 20–24 years [11]. The ages of those examined ranged from 13 to 55 years and averaged 25.8 years. There were 142 girls aged 13 to 17 years, representing 7.85%. The WHO defines pregnancy up to and including the age of 19 years as adolescent pregnancy [11]. Hence, we additionally defined for further analysis the subgroup of pregnant women aged 13–19 years, featuring 378 individuals (20.88% of the total group). The age distribution of pregnant women in the study group is presented in Figure 1.

The group of male partners of pregnant women featured 481 patients. These included individuals who reported sexual contacts with both people living with HIV (PLHIV) and with pregnant wives in the same period of time, potentially representing a source of infection for future mothers and children. The ages of males ranged from 15 to 60 years (mean age 29.05 ± 11.99 years). Males aged between 15 and 17 years comprised 13.33% of the total.

### 2.1. HCV Serology

Markers of HCV infection (anti-HCV total) were determined by ELISA. Testing was carried out in duplicate using diagnostic kits manufactured by Diagnostic Systems RPC (Nizhny Novgorod, Russia) and Vector-Best CJSC (Novosibirsk, Russia), in compliance with the manufacturer’s instructions. 

### 2.2. HCV RNA Extraction, Amplification, and Sequencing

For HCV RNA detection, nucleic acids were extracted from blood plasma using the ‘AmpliPrime Ribo-Prep’ commercial kit. The next step was reverse transcription using a reagent set for obtaining cDNA from RNA template, namely ‘REVERTA-L’ (CRIE, Moscow, Russia). Viral presence test was executed by real-time polymerase chain reaction (PCR) with hybridization fluorescence detection. First, all samples were tested using the ‘AmpliSens^®^ HCV-FL’ commercial kit (CRIE, Moscow, Russia); the sensitivity of the test system was 100 IU/mL. Viral loads were determined using ‘AmpliSense HCV-monitor-FL’ (CRIE, Moscow, Russia) reagents according to manufacturer instructions. Sequencing of HCV gene nucleotide sequences was performed only when viral loads exceeded 1000 IU/mL. Viral genotypes were determined using specific primers for a region of the HCV *NS5b* gene. Specific primers for each genotype were then used to obtain sequences of three regions of the virus, specifically those in which mutations are associated with resistance (*NS3, NS5A, NS5B*).

Amplification products were purified and evaluated for fragment length and concentration. Determination of nucleotide sequences was carried out using an ABI Prism 3500 genetic analyzer (Applied Biosystems, Bedford, MA, USA) according to manufacturer instructions. Primary analysis of the obtained consensus nucleotide sequences was carried out using the NCBI BLAST program in comparison with the nucleotide sequences present in the GenBank international database. The resulting sequences were submitted to the GenBank international database, where they were assigned the corresponding numbers (OR902095-OR902105 (*NS5B*), OR917720-OR917730 (*NS5A*), OR917741-OR917751 (*NS3*)).

### 2.3. Data Analysis

Primary analysis of the obtained fragments was performed using the BLAST algorithm (https://blast.ncbi.nlm.nih.gov/, accessed on 11 December 2023) on the nucleotide sequences provided in the GenBank sequence database. The resulting sequences were aligned in the MEGAv.11 program using the ClustalW algorithm. Phylogenetic trees were constructed using the neighbor-joining method; the significance of the tree was assessed using bootstrap analysis with 1000 replicates. Fragments of regions *NS3*, *NS5A*, and *NS5B* were assessed for the presence of nucleotide substitutions leading to drug-resistance mutations using the Genafor Geno2pheno HCV tool (https://hcv.geno2pheno.org/index.php, accessed on 11 December 2023).

### 2.4. Statistical Analysis

Statistical data processing was carried out using the Excel (Microsoft Corp., Redmond, WA, USA) and Prizm 5.0 (GraphPad Software, Inc., La Jolla, CA, USA) software packages. The “exact” Clopper–Pearson interval was used to estimate statistical uncertainty. Results are represented as frequencies (percentages) with 95% confidence interval (95% CI). Depending on sample characteristics, the Fisher exact test or Yates-corrected chi-squared test was used to evaluate the statistical significance of numeric data obtained during paired comparison. A probability value of *p* < 0.05 was taken as the statistical significance threshold.

### 2.5. Ethics Statement

All procedures performed in studies involving participating human subjects were performed in accordance with the ethical standards of the institution and/or national research committee and with the 1964 Helsinki Conference declaration and its subsequent amendments or comparable ethical standards. This study was approved by the Ethics Committee of the Saint Petersburg Pasteur Institute and by the Ethics Committee of Guinea (Minutes No. 129/CNERS/16, 31 August 2015). All examined individuals gave their written informed consent for participation in the study.

## 3. Results

In the course of this study, HCV antibodies were detected in 58 of the pregnant women examined (3.2%, 95% CI: 2.44–4.12%) and in 16 male partners (3.33%, 95% CI: 1.91–5.35%). Using PCR, HCV RNA was detected in nine cases in women (0.5%, 95% CI: 0.23–0.94%) and in all the anti-HCV-positive male partners (3.33%, 95% CI: 1.91–5.35%). HCV RNA was more common in the men than in the pregnant women (χ^2^ = 25.6, df 1, *p* < 0.0001, RR = 6.69 with 95% CI: 2.97–15.04).

The prevalence of anti-HCV and HCV RNA in the pregnant women was determined in rural and urban regions (Table 1), as well as in cities of residence (Table 2).

Hepatitis C antibodies were significantly more frequent in Pita compared to Kindia (χ^2^ = 16.684, df 1, *p* < 0.0001), Conakry (χ^2^ = 35.012, df 1, *p* < 0.0001), and Cancan (χ^2^ = 6.893, df 1, *p* = 0.0087). Antibodies were significantly more frequent in Faran compared to Kindia (χ^2^ = 21.042, df 1, *p* < 0.0001), Conakry (χ^2^ = 34.564, df 1, *p* < 0.0001), and Kankan (χ^2^ = 6.67, df 1, *p* = 0.0098). Antibodies were also significantly more frequent in Macenta compared to Conakry (χ^2^ = 11.565, df 1, *p* = 0.0007). There were no significant differences between six cities in terms of HCV RNA prevalence (*p* > 0.05).

The occurrences of anti-HCV and HCV RNA were analyzed according to the familial status of the pregnant women (Table 3). There were no significant differences between groups of different marital status (women) in terms of anti-HCV/ HCV RNA prevalence (*p* > 0.05).

The prevalence of anti-HCV and HCV RNA was analyzed according to the women’s occupation (Table 4). There was some trending towards higher anti-HCV frequencies among hairdressers and nurses, but no significant differences were found (*p* > 0.05).

The prevalence of anti-HCV and HCV RNA depending on the age of the pregnant women was assessed. The data on the age distribution of the patients with HCV markers detected are presented in Table 5.

There were no significant differences in the prevalence of anti-HCV among the pregnant women by age group. There was a significant predominance of HCV RNA occurrence in the group aged 13–17 years compared to the women who were 18–35 years old (*p* = 0.0258, RR = 6.325 with 95% CI: 1.527–26.203).

The HCV viral load was determined for all the RNA-HCV-positive samples. The HCV viral load exceeded 1000 IU/mL in all the women (100%, 95% CI: 66.37–100%) and only in two cases in the men (12.5%, 95% CI: 1.55–38.35%) at *p* < 0.0001. 

**Figure 2:**
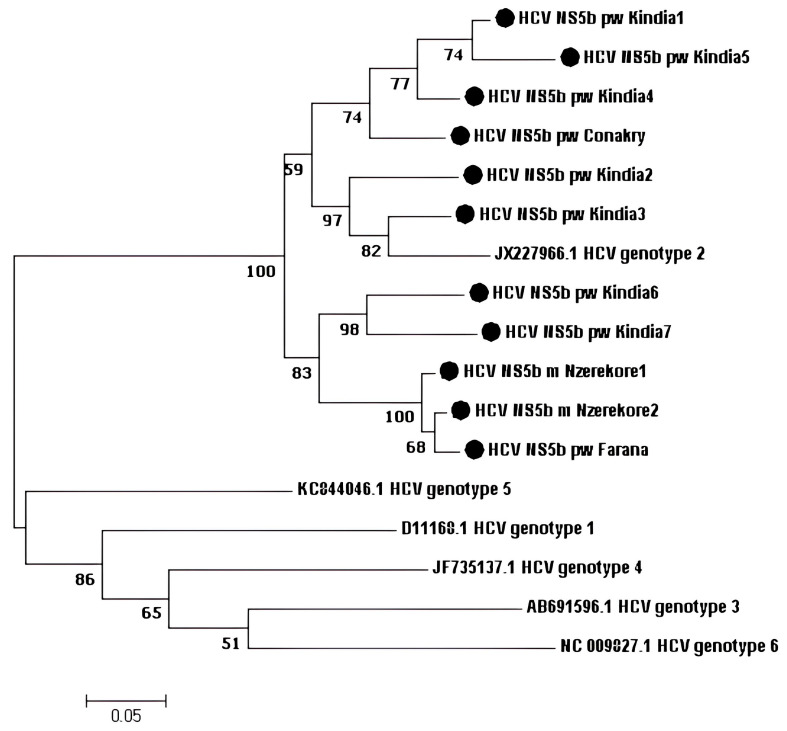
Phylogenetic analysis of the nucleotide sequences of the HCV *NS5B* gene isolated from hepatitis C patients from the Republic of Guinea in comparison with reference sequences present in GenBank. Reference sequences indicating genotype are designated by GenBank codes. Samples studied in this work are designated by circle.

The HCV genes *NS5A* and *NS5B* and the *NS3* gene fragment were sequenced for 11 samples. To determine the viral genotype, a phylogenetic analysis of the NS5B gene nucleotide sequences was performed using the GenBank database reference sequences for genotypes 1–6 (Figure 2).

**Figure 3:**
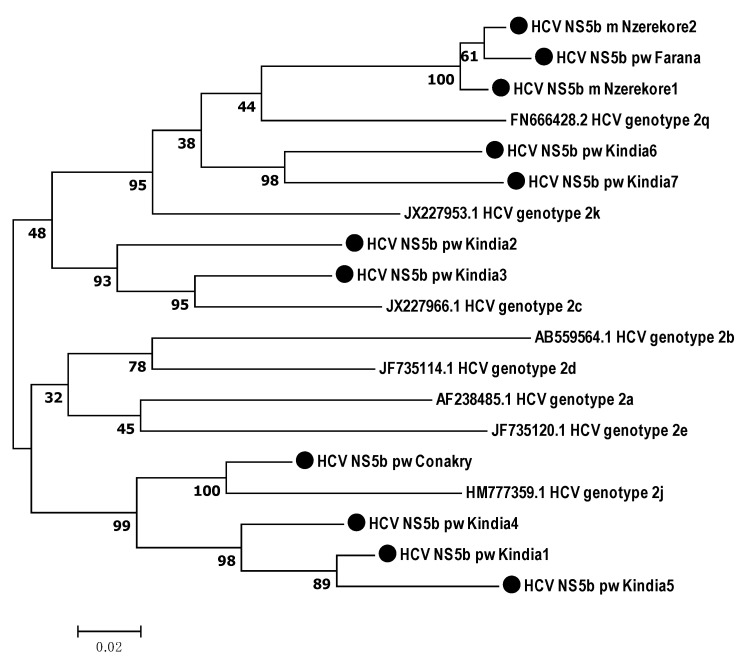
Phylogenetic analysis of the nucleotide sequences of the HCV *NS5B* gene isolated from hepatitis C patients from the Republic of Guinea in comparison with reference sequences with known subgenotype present in GenBank. Reference sequences indicating genotype are designated by GenBank codes. Samples studied in this work are designated by circle.

**Figure 4:**
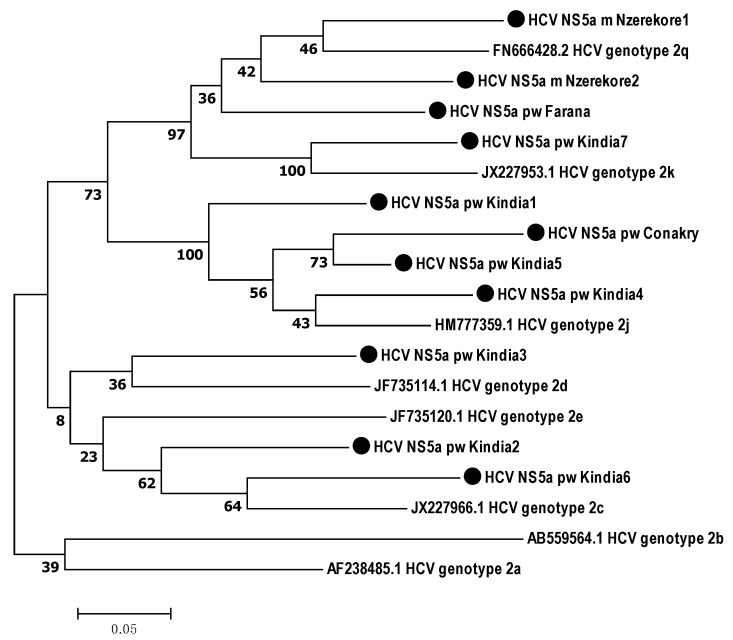
Phylogenetic analysis of the nucleotide sequences of the HCV *NS5A* gene isolated from hepatitis C patients from the Republic of Guinea compared with the sequences of different genotype 2 subtypes reported in GenBank. Reference sequences indicating genotype are designated by GenBank codes. Samples studied in this work are designated by circle.

**Figure 5:**
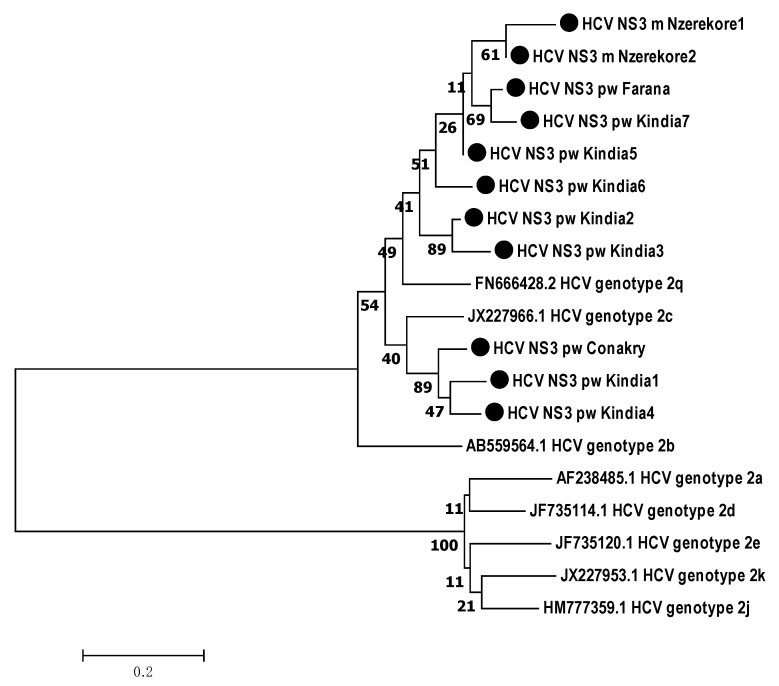
Phylogenetic analysis of the nucleotide sequences of the HCV *NS3* gene isolated from hepatitis C patients from the Republic of Guinea compared with the sequences of different genotype 2 subtypes reported in GenBank. Reference sequences indicating genotype are designated by GenBank codes. Samples studied in this work are designated by circle.

According to the phylogenetic analysis, all the samples obtained from the Guinean residents belonged to genotype 2. Therefore, HCV target regions were analyzed in relation to genotype 2 subtypes (Figure 3, Figure 4 and Figure 5). 

According to the phylogenetic analysis of the *NS5B* gene nucleotide sequences, five isolates (45.45%, 95% CI: 16.75–76.62%) were closest to subtype 2q from the following participants: two males from Nzerekore; one female from Farana; and two females from Kindia. Two samples (18.18%, 95% CI: 2.28–51.78%) from Kindia were assigned to 2c. Four isolates (36.36%, 95% CI: 10.93–69.21%) were phylogenetically close to genotype 2j (Figure 3).

According to the phylogenetic analysis of the *NS5A* gene nucleotide sequences, three isolates were closest to subtype 2q, including those from two males from Nzerekore and one female from Farana. Four isolates were phylogenetically close to genotype 2j, two samples from Kindia were assigned to 2c, and one sample from Kindia was determined to be 2d (Figure 4).

According to the phylogenetic analysis of the *NS3* gene nucleotide sequences, eight samples were close to subtype 2q, and three samples were close to subtype 2c (Figure 5).

Results of phylogenetic analysis of HCV genomic regions was summarized in Table 6.

When the samples were genotyped using the Genafor Geno2pheno HCV genotyping tool (based on *NS5B, NS5A, NS3* regions), all the isolates were also assigned to genotype 2, and the majority were assigned the same subtype as in the phylogenetic analysis. However, the results of the subtype determination differed for some samples. Thus, in the NS5B region analysis, the HCV_NS5b_pw_Kindia5 and HCV_NS5b_pw_Kindia7 samples, which were phylogenetically assigned to subtypes 2j and 2q, respectively, were identified as 2c and 2k. When the *NS5A* region was analyzed, two isolates that were phylogenetically assigned to subtype 2c (HCV_NS5a_pw_Kindia2, HCV_NS5a_pw_Kindia6) were found to be close to subtype 2f. When analyzing the NS3 region, only three samples had the same subtypes identified in the phylogenetic analysis. For another five samples, the subtype matched the one identified in the phylogenetic analysis of the *NS5B* and/or NS5A regions. In three cases, the most genetically close reference was a sample whose identity was difficult to determine and was labelled as “2 × 3” in Genafor Geno2pheno HCV. Table 7 shows the results of the genotyping by the *NS5B, NS5A, NS3* regions according to Genafor Geno2pheno HCV.

**Figure 6:**
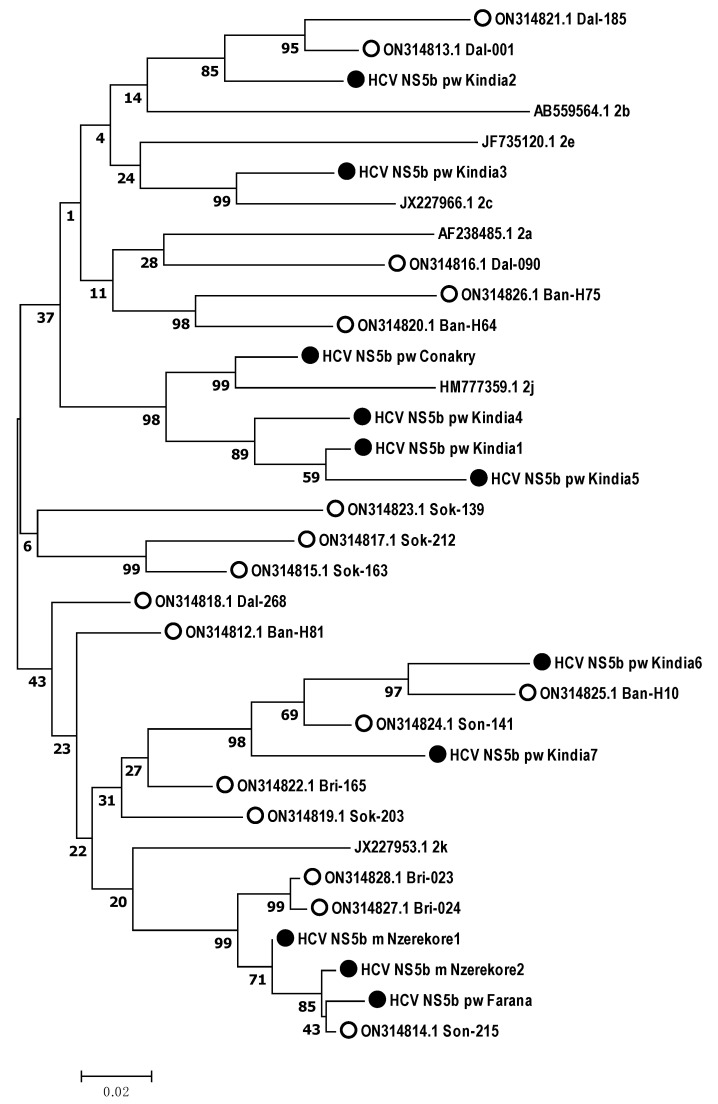
Phylogenetic analysis of the nucleotide sequences of the HCV *NS5B* gene isolated from hepatitis C patients from the Republic of Guinea compared with the sequences of different genotype 2 subtypes reported in GenBank, and samples previously identified in the country. Reference sequences indicating genotype are designated by GenBank codes. Samples from the Republic of Guinea are indicated by circles. Samples identified in this study are indicated by black circles.

**Figure 7:**
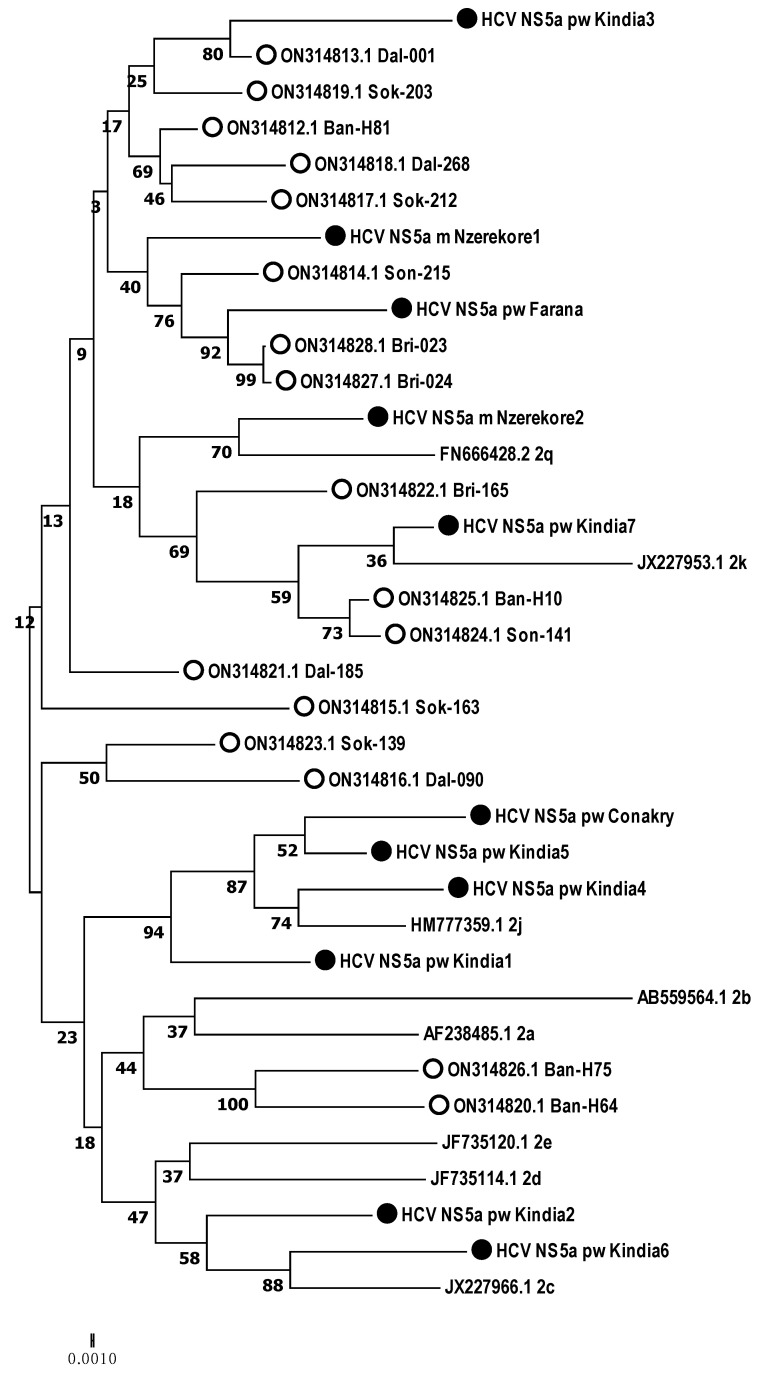
Phylogenetic analysis of the nucleotide sequences of the HCV *NS5A* gene isolated from hepatitis C patients from the Republic of Guinea compared with the sequences of different genotype 2 subtypes reported in GenBank, and samples previously identified in the country. Reference sequences indicating genotype are designated by GenBank codes. Samples from the Republic of Guinea are indicated by circles. Samples identified in this study are indicated by black circles.

**Figure 8:**
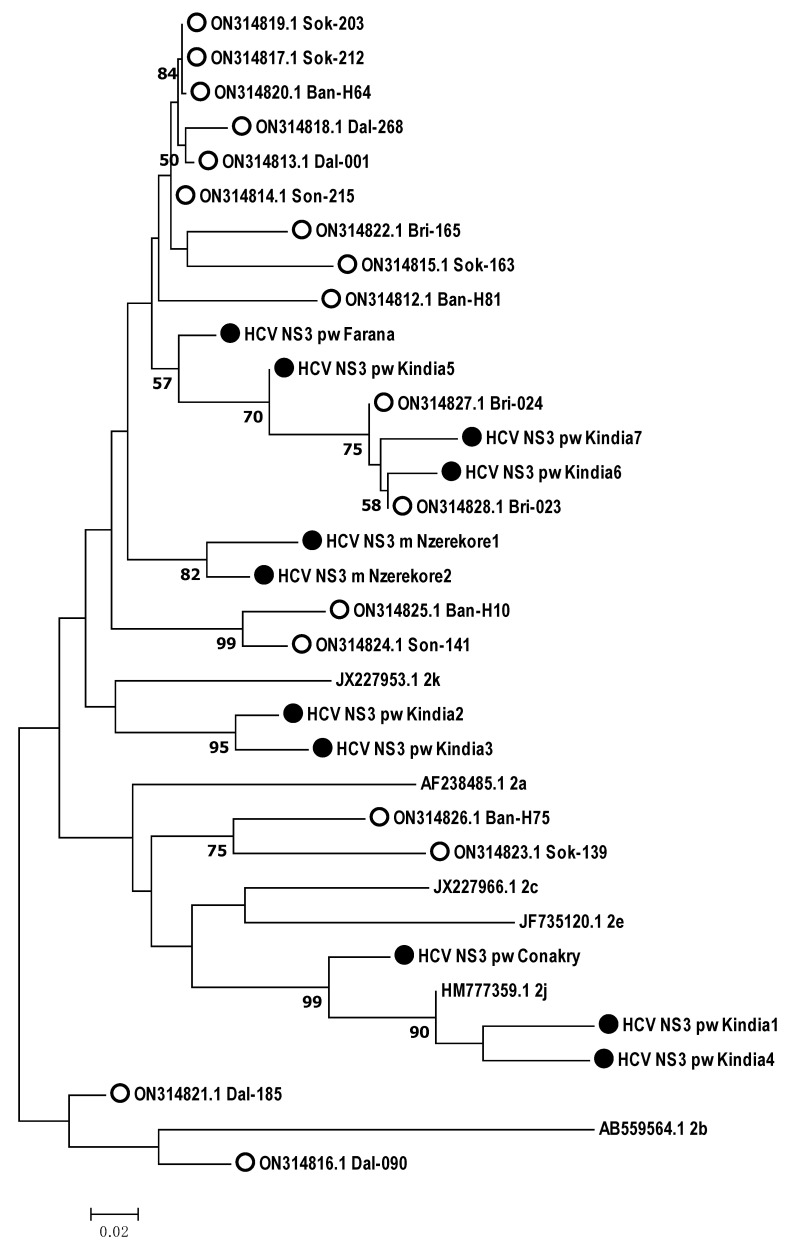
Phylogenetic analysis of the nucleotide sequences of the HCV *NS3* gene isolated from hepatitis C patients from the Republic of Guinea compared with the sequences of different genotype 2 subtypes reported in GenBank, and samples previously identified in the country. Reference sequences indicating genotype are designated by GenBank codes. Samples from the Republic of Guinea are indicated by circles. Samples identified in this study are indicated by black circles.

Additionally, genomic fragments of identified HCV isolates were phylogenetically analyzed in comparison with recently described samples from the Republic of Guinea [12] (Figure 6, Figure 7 and Figure 8).

No drug resistance mutations could be identified among the isolates studied. However, polymorphic variants different from the wild-type virus were identified. In the HCV_pw_Farana sample, an uncharacterized 289L amino acid substitution was identified in the *NS5B* region at position 289L, for which a mutation is known to cause resistance of the virus to sofosbuvir.

## 4. Discussion

The epidemiology of viral hepatitis is a dynamic phenomenon, subject to change due to socioeconomic development, sociocultural practices, public interventions through national programs, and the evolving understanding of hepatitis viruses. There have been tremendous advances in the understanding of viral hepatitis, changes in the understanding of chronic and acute forms of disease caused by hepatotropic viruses, the development of methods to detect viruses in early stages or latent forms of the disease, and the development of antiviral drugs and effective vaccines against some viruses.

Before proceeding directly to a discussion of the results, we would like to draw attention to a number of limitations of this study, primarily due to its small scale as a pilot project. First of all, our attention was focused on patients without a history of markers of other blood-borne infections, i.e., conditionally healthy patients. This group is characterized by a reduced probability of HCV detection and, therefore, the results of this study are highly representative. However, we cannot deny the fact that we excluded from attention the groups of patients in which the probability of HBV detection is much higher, which, accordingly, did not allow us to fully assess the prevalence of HCV infection in the population. 

Furthermore, this study did not investigate social risks in relation to HCV infection. Within the framework of this work, we did not have the resources necessary for a full-fledged study of social risks in the patients’ anamnesis. We therefore limited ourselves to the basic information provided by the patients during examination in medical and preventive institutions.

HCV antibodies were detected in 3.2% of the individuals examined and HCV RNA in only 0.5%, well below the average prevalence in Africa (approximately 7%) [4]. Nevertheless, HCV prevalence in the Republic of Guinea is relatively low, with the incidence of HCV antibodies among women in 2004–2009 being approximately 0.9% [13]. However, our finding is broadly consistent with the calculated HCV prevalence estimates of 0.72% in Southern Africa, 3.0% in East Africa, 4.14% in West Africa, and 7.82% in Central Africa [14]. Since blood donors have the lowest documented HCV prevalence (1.78%), followed by pregnant women (2.51%), PLHIV (3.57%), the general population (5.41%), and various high-risk groups (>10%) [15,16], the low HCV prevalence we show in pregnant women compared to the conventionally healthy population and at-risk groups seems legitimate. The prevalence of anti-HCV among pregnant women in the Brong Ahafo region of Ghana was 12% [17], which is significantly higher than in our study. This discrepancy may be due to the different levels of prevalence of the virus among women in Ghana (HCV: 3.2%) [15,16]. The prevalence of HCV among pregnant women in Côte d’Ivoire was 0.8% in 2004 [18], and it was 0.2% in Mali in 2008–2009, rising significantly in older women (6.1%) [19]. 

In Guinea-Bissau, the prevalence of anti-HCV in women was 0.6% [20], which is broadly in line with our results. The similarities and differences in the results of HCV detection in pregnant women observed in different countries of the subregion may be related to geographical features, the diversity of samples and screening methods, as well as cultural and behavioral differences with regard to risk factors, transmission patterns dictated by sociocultural practices, and environmental factors. Note that according to WHO estimates, only 6% of people infected with HCV in African countries know their serologic status [21]. The higher incidence of bloodborne infections in men than in women is easily explained by the freer and riskier behavior of men, including sexual behavior. This is characteristic of most regions of the world.

The present study evaluated the possible relationship between serological and molecular markers of HCV infection (prevalence) and other factors, as follows: pregnant women’s region of residence; urban or rural area; occupation; and age. Significant differences in anti-HCV prevalence between cities were shown, suggesting that residents of Pita, Farana, and Macenta were more likely to be exposed to the virus, but no significant differences were found for HCV RNA. It is possible that the significant differences in antibody prevalence were due to differences in the size of the study groups from each city. There were no significant differences in the prevalence of anti-HCV among pregnant women by age group. There was a significant predominance of HCV RNA occurrence in the group aged 13–17 years compared to women who were 18–35 years old (*p* = 0.0258, RR = 6.325 with 95% CI: 1.527–26.203). No significant differences related to other factors were found.

The HCV genome is represented by a single-stranded, positive-sense RNA approximately 9600 nt in length, containing highly conserved untranslated regions (UTRs), 5’-UTR and 3’-UTR, flanking a single open reading frame comprising 9030–9099 nt (depending on viral genotype) encoding a single precursor polyprotein 3010–3033 amino acid residues in length [22]. HCV non-structural proteins are multifunctional, participating in particle morphogenesis, RNA replication, and the regulation of cell functions. They are of particular interest as potential antiviral drug targets due to their basic functions. For example, *NS5B* is an RNA-dependent RNA polymerase that initiates HCV negative-strand RNA synthesis, playing a key role in viral replication [22]. The genotypic diversity of HCV is represented by eight genotypes (1–8), among which the differences in full nucleotide sequences are approximately 31–33%. There are also 100 sub-genotypes (defined for genotypes 1–4 and 6–7, the number of which is constantly increasing), the difference between which can reach 15–25% [23]. Almost 30–40% of all HCV infections are of genotype 1, while genotype 3 is present in 22% of cases. Genotypes 2, 4, 5, and 6 account for approximately 9–13%, 8%, 1%, and 6% of cases, respectively [4]. Genotypes 7 and 8 are not common, occurring in less than 1% of cases [23]. HCV genotypes and subtypes differ with respect to the natural course of infection, transmission routes, disease progression, clinical outcomes, effective treatment regimens, and responses to antiviral therapy [24].

All the HCV isolates identified in this study were categorized as genotype 2. Different geographical regions are characterized not only by different frequencies of HCV in general and chronic hepatitis C in particular, but also by the variability of the viral genetic variants represented. Thus, although genotypes 1–3 are detected worldwide, genotype 1 (primarily subtypes 1a and 1b) is the most common in Russia, USA, Japan, and European countries. Genotype 2 is widespread in Russia and some European countries, as well as in West Africa, with subtype 2b predominating in Northern Europe and subtype 2c in Southern Europe. Genotype 3 (subtype 3a) is predominant in Nepal, Pakistan, and India. Genotype 4 is more common in North Africa and the Middle East, while genotypes 5 and 6 are more common in Hong Kong and South Africa. Genotype 7 is represented in the Democratic Republic of Congo, and genotype 8 has been determined only in India [4,23]. It should be noted that the genotypic profile of a region can change rapidly due to the introduction of rare genetic variants characteristic of other countries, due to demographic and social factors, including international migration due to labor and tourism [25]. 

HCV genotype 2 is characteristic of West African countries, where more than ten sub-genotypes of this genetic variant have been described. For example, in a meta-analysis of PLHIV, the HCV RNA prevalence in West Africa averaged 2%, in Senegal and Côte d’Ivoire 1.5–3.5%, and in Mali and Guinea-Bissau less than 1.5%, with only genotype 2 detected in Gambia and Guinea-Bissau. In Ghana, in addition to HCV 2, subtype 1b was detected in much smaller numbers [26,27]. The detection of four different sub-genotypes (c, j, k, q), and an undetectable variant of genotype 2 among only eleven identified HCV isolates, is alarming. However, a variety of subtypes have been shown for this region: variants 2a, 2b, and 2c are common in Guinea-Bissau, Ghana, Benin, Burkina Faso, Gambia, and Senegal [28]. A recent study in the Republic of Guinea identified variants 2k and 2q, isolates close to 2j and 2m, and also identified nine sequences of previously unknown subtypes [12]. Thus, the diversity of HCV genotype 2 in the surveyed group confirms and complements the known data on the variability of this pathogen in the country. 

The absence of MDR was probably due to the low representation of the virus in the region, as well as to the almost complete absence of therapy aimed at the treatment HCV-infected persons in the country. In addition, it should be taken into account that not all amino acid substitutions leading to pharmacoresistance to certain groups of drugs are currently known. New data are frequently appearing, which increases the list of significant amino acid substitutions. This is due to the fact that, although high genetic heterogeneity is characteristic of all RNA viruses, including HCV, the issue of frequently occurring polymorphic variants that could potentially be the cause of decreased sensitivity or complete resistance to certain drugs has not been fully studied. Assessment of the heterogeneity of nucleotide sequences of pharmacoresistance-associated fragments of the viral genome plays a significant role, even in cases in which MDRs have not been detected, as it is important to analyze natural polymorphic patterns that could potentially be MDRs. 

With the emergence of new drugs and new treatment regimens, it cannot be excluded that frequent substitutions not previously identified as resistance-related will be found to be so. In addition, substitutions that represent a natural polymorphism for one viral genotype may turn out to be drug-resistance mutations for another. Moreover, the ways in which these mutations interact with already known polymorphic variants associated with resistance can be complex. It is theoretically possible that mutation/polymorphism combinations could provide a new resistance variant, exacerbate existing resistance, or, conversely, increase the efficacy of antiviral drugs. Some polymorphic variants may in fact be clinically significant, leading to future viral resistance due to therapy or other factors, but ongoing monitoring and data collection are needed to understand this. In this regard, the diversity of HCV genotype 2 subtypes in African countries is of great interest, especially since a large number of natural polymorphisms have been identified in the genomic regions analyzed. 

It is interesting to note that for some of the HCV isolates we identified in this study, those described earlier in this country were the most genetically similar, but the differences were sufficiently large for the sequences not to be clustered. It can be hypothesized that the sample collection was not sufficiently complete to represent the full diversity of HCV genotype 2 in the Republic of Guinea or to find intermediate forms, crossovers, and routes of transmission. All this suggests the need for more extensive studies of HCV in the Republic of Guinea.

## 5. Conclusions

This study is an important pilot project to detect HCV in pregnant women in the Republic of Guinea. The results showed a high prevalence of HCV markers in patients without a history of markers for other blood-borne infections, which emphasizes the need to focus on pregnant women as a special risk group for HCV. Even though Guinea is a relatively benign area in terms of HCV prevalence, there is strong evidence that the project should be further developed and expanded to other groups of pregnant women and their partners.

## Figures and Tables

**Table 1:** Prevalence of anti-HCV and HCV RNA among pregnant women from urban and rural areas.

Region	Proportion of Those Examined with Anti-HCV/HCV RNA	
	Anti-HCV (%, 95% CI)	HCV RNA (%, 95% CI)
Rural regions	4.23% (2.48–6.68%)	0% (0–0.91%)
Urban regions	2.91% (2.1–3.93%)	0.64% (0.29–1.21%)

**Table 2:** Prevalence of anti-HCV and HCV RNA among pregnant women from six cities.

City	Proportion of Those Examined with Anti-HCV/HCV RNA	
	Anti-HCV (%, 95% CI)	HCV RNA (%, 95% CI)
Kindia	2.92% (2.08–3.99%)	0.54% (0.22–1.11%)
Conakry	0.33% (0.01–1.81%)	0.33% (0.01–1.81%)
Kankan	1.39% (0.04–7.50%)	0% (0–4.99%)
Farana	14.75% (6.98–26.17%)	1.64% (0.04–8.8%)
Macenta	8.33% (1.75–22.47%)	0% (0–9.74%)
Pita	16.67% (6.37–32.81%)	0% (0–9.74%)

There were no significant differences between urban and rural areas in anti-HCV or HCV RNA prevalence (*p* > 0.05).

**Table 3:** Prevalence of anti-HCV and HCV RNA among pregnant women by marital status.

Marital Status	Proportion of Those Examined with Anti-HCV/HCV RNA	
	Anti-HCV (%, 95% CI)	HCV RNA (%, 95% CI)
Married	2.61% (1.7–3.83%)	0.31% (0.06–0.91%)
Single	4.06% (2.59–6.04%)	0.71% (0.19–1.8%)
Single mother	3.53% (1.71–6.4%)	0.71% (0.09–2.53%)

**Table 4:** Prevalence of anti-HCV and HCV RNA among pregnant women by occupation.

Occupation	Proportion of Those Examined with Anti-HCV/HCV RNA	
	Anti-HCV (%, 95% CI)	HCV RNA (%, 95% CI)
Midwife	4.62% (0.96–12.9%)	0% (0–5.52%)
Housewife	2.58% (1.51–4.1%)	0.3% (0.04–1.09%)
Nurse	6.82% (92.54–14.25%)	2.27% (0.28–7.97%)
Hairdresser	6.15% (1.7–15.01%)	0% (0–5.52%)
Teacher	1.85% (0.05–9.89%)	0% (0–6.6%)
Saleswoman	3.59% (2.14–5.62%)	0.2% (0.01–1.11%)
Seamstress	0% (0–10.58%)	0% (0–10.58%)
Student	4.11% (0.86–11.54%)	1.37% (0.03–7.4%)
Learner	2.21% (0.81–4.74%)	1.1% (0.23–3.19%)

**Table 5:** Prevalence of anti-HCV and HCV RNA among pregnant women by age.

Age Group	Proportion of Those Examined with Anti-HCV/HCV RNA	
	Anti-HCV (%, 95% CI)	HCV RNA (%, 95% CI)
13–17 years old	1.41% (0.17–5%)	2.11% (0.44–6.05%)
13–19 years old	2.38% (1.09–4.47%)	0.79% (0.16–2.3%)
20–24 years old	3.32% (1.87–5.41%)	0.44% (0.05–1.59%)
18–35 years old	3.65% (2.72–4.68%)	0.33% (0.11–0.78%)
≥36 years old	1.17% (0.14–4.16%)	0% (0–2.13%)

**Table 6:** Genotypes of HCV isolates from the Republic of Guinea determined by phylogenetic analysis of *NS5B*, *NS5A*, and *NS3* regions.

Sample	Subtype, According to HCV Region Analysis		
	NS5B	NS5A	NS3
HCV_m_Nzerekore1	2q	2q	2q
HCV_m_Nzerekore2	2q	2q	2q
HCV_pw_Farana	2q	2q	2q
HCV_pw_Conakry	2j	2j	2c
HCV_pw_Kindia1	2j	2j	2c
HCV_pw_Kindia2	2c	2c	2q
HCV_pw_Kindia3	2c	2d	2q
HCV_pw_Kindia4	2j	2j	2c
HCV_pw_Kindia5	2j	2j	2q
HCV_pw_Kindia6	2q	2c	2q
HCV pw_Kindia7	2q	2k	2q

**Table 7:** Genotypes of HCV isolates from the Republic of Guinea based on specific region analysis (NS5B, NS5A, NS3) using the Genafor Geno2pheno HCV tool.

Sample	NS5B			NS5A			NS53		
	Closest Reference	Genotype	Identity	Closest Reference	Genotype	Identity	Closest Reference	Genotype	Identity
HCV_m_Nzerekore1	FN666428	2q	87.21%	FN666428	2q	89.81%	FN666428	2q	85.82%
HCV_m_Nzerekore2	FN666428	2q	87.04%	FN666428	2q	89.97%	FN666428	2q	85.64%
HCV_pw_Farana	FN666428	2q	86.81%	FN666428	2q	86.83%	FN666428	2q	85.45%
HCV_pw_Conakry	HM777358	2j	90.95%	HM777358	2j	86.36%	HM777358	2j	92.08%
HCV_pw_Kindia1	HM777358	2j	85.8%	HM777358	2j	88.23%	HM777358	2j	93.19%
HCV_pw_Kindia2	D50409	2c	87.72%	KC844042	2f	84.19%	D50409	2c	85.27%
HCV_pw_Kindia3	D50409	2c	90.78%	D50409	2c	84.98%	D50409	2c	85.45%
HCV_pw_Kindia4	HM777358	2j	86.53%	HM777358	2j	88.7%	HM777358	2j	93.55%
HCV_pw_Kindia5	D50409	2c	86.13%	HM777358	2j	87.3%	JF735118	2 × 3	84.16%
HCV_pw_Kindia6	FN666428	2q	86.13%	KC844042	2f	84.33%	JF735118	2 × 3	84.53%
HCV pw_Kindia7	AB031663	2k	86.3%	AB031663	2k	85.76%	JF735118	2 × 3	85.27%

## Data Availability

Data available on request from the authors.
